# Treatment Options of First-Line Tyrosine Kinase Inhibitors and Subsequent Systemic Chemotherapy Agents for Advanced EGFR Mutant Lung Adenocarcinoma Patients: Implications From Taiwan Cancer Registry Cohort

**DOI:** 10.3389/fonc.2020.590356

**Published:** 2021-01-08

**Authors:** Sheng-Kai Liang, Li-Ta Keng, Chia-Hao Chang, Yueh-Feng Wen, Meng-Rui Lee, Ching-Yao Yang, Jann-Yuan Wang, Jen-Chung Ko, Jin-Yuan Shih, Chong-Jen Yu

**Affiliations:** ^1^Department of Internal Medicine, National Taiwan University Hospital Hsinchu Branch, Hsinchu City, Taiwan; ^2^Institute of Biotechnology, National Tsing Hua University, Hsinchu City, Taiwan; ^3^Department of Internal Medicine, National Taiwan University Hospital and College of Medicine, National Taiwan University, Taipei City, Taiwan

**Keywords:** lung adenocarcinoma, gefitinib, erlotinib, afatinib, epidermal growth factor receptor mutation, subsequent therapy

## Abstract

**Objectives:**

Large-scale, population-based real-world studies on the treatment outcomes of first-line tyrosine kinase inhibitors (TKIs) and subsequent systemic chemotherapy agents for lung adenocarcinoma (with activating epidermal growth factor receptor [EGFR] mutations) remain limited.

**Materials and Methods:**

From March 2014 to December 2016, patients with advanced lung adenocarcinoma, identified from the Taiwan Cancer Registry were included in this study if they received any of the three TKIs as first-line treatment. The primary outcome was overall survival (OS). The secondary outcome was time-to-treatment discontinuation (TTD).

**Results:**

A total of 4,889 patients (median age: 67 years and two-thirds with distant metastasis) were recruited (1,778 gefitinib, 1,599 erlotinib, and 1,512 afatinib users). A 1:1 propensity score (PS)-matched cohorts of 1,228 afatinib/erlotinib and 1054 afatinib/gefitinib was created. After PS matching, it was found that afatinib was not associated with better OS (afatinib vs. erlotinib, HR: 0.96, 95% CI: 0.86–1.07; afatinib vs. gefitinib, HR: 0.91, 95% CI: 0.81–1.02). In the subgroup analysis, afatinib demonstrated a survival benefit in patients with active smoking (afatinib vs. erlotinib, HR: 0.69, 95% CI: 0.51–0.93; afatinib vs. gefitinib, HR: 0.67, 95% CI: 0.48–0.94) and ECOG > 1 (afatinib vs. erlotinib, HR: 0.79, 95% CI: 0.63–0.99; afatinib vs. gefitinib, HR: 0.78, 95% CI: 0.62–0.98). A total of 41.1% (n = 1992) of first-line TKI users received subsequent chemotherapy. Among the three TKI groups, pemetrexed usage was associated with better OS compared with other chemotherapy agents, with the exception of gemcitabine in the afatinib and gefitinib groups. Pemetrexed and gemcitabine had the longest TTD of 3–4 months.

**Conclusions:**

Among patients with *EGFR* mutant lung adenocarcinoma, afatinib use may not provide longer OS compared with first-generation TKIs. Afatinib may be preferably considered among patients with active smoking and should not be withheld among those with worse performance status. With 40% of patients receiving subsequent chemotherapy, pemetrexed may be the preferred agent, while gemcitabine can be a reasonable alternative.

## Introduction

Lung cancer is the leading cause of cancer-related deaths in the 21^st^ century (1, 2). Adenocarcinoma is the major histological type of non-small cell lung cancer (NSCLC), but the standard care for patients with metastatic NSCLC has shifted from traditional platinum-based doublets to precision targeted therapy to the driver genes with mutations, such as the epidermal growth factor receptor (*EGFR)*, anaplastic lymphoma kinase (*ALK*), *ROS-1*, and *BRAF* (3, 4). Targeting lung adenocarcinoma with *EGFR* mutations among Asians is important because they have a significantly higher prevalence of the *EGFR* mutation compared with the Caucasians (5–7). Multiple generations of EGFR tyrosine kinase inhibitors (TKIs) have been effective as first-line therapy for advanced *EGFR*-mutant lung adenocarcinoma patients (8–12).

Gefitinib, erlotinib, and afatinib are widely prescribed first-line TKIs worldwide. All of them provide robust and similar effects in advanced *EGFR*-mutant lung adenocarcinoma patients (11, 13, 14). Although afatinib has minimal clinical significance in progression-free survival (PFS) compared with gefitinib (median PFS 11.0 vs. 10.9 months, respectively) in the first-line setting (15), it did not show improved overall survival (OS) compared with gefitinib (13, 14). Recently, several real-world studies have investigated the characteristics and clinical effectiveness of these three EGFR TKIs administered in advanced lung adenocarcinoma patients (16–20). However, the conclusions from these studies may not provide convincing evidence for clinical practice because of their limited case numbers, discrepant recruitment time, disproportional populations in which TKIs were used, and lack of information on subsequent therapy after first-line EGFR TKI failure (16–20).

Prolonging cancer patients’ OS is a major goal of all cancer treatments, and understanding the optimal treatment sequences is a key factor that allows patients to live longer. p.T790M in the *EGFR* gene is the most common acquired resistance mechanism following first-line TKI treatment (21), and osimertinib proved to be effective in patients with the *EGFR* p.T790M mutation as a standard second-line treatment (22). Owing to the unavailability of osimertinib in some situations, for cases without acquired p.T790M or accessible tumor tissues for re-biopsy, chemotherapy remains an important subsequent therapy after first-line TKI (23, 24). Furthermore, only, few studies have investigated the optimal regimen of chemotherapy as second-line treatment in patients who are p.T790M negative or have an unknown acquired resistance mechanism after first-line TKI failure (25, 26).

This study, therefore, aimed to investigate the treatment sequences and clinical outcomes of treatment-naïve, EGFR-mutant advanced lung adenocarcinoma patients receiving TKIs in a real-world, population-based setting. Additionally, we explored the prognostic factors of TKI users and treatment durations of individuals after they underwent subsequent chemotherapy. Our results were informative with respect to clinical decision-making.

## Materials and Methods

### Ethics Statement

This study was approved by the Institutional Review Board (IRB) committee of the National Taiwan University Hospital Hsinchu Branch (NTUH-HC REC: 105-040-E). The IRB waived the requirement of informed consent because the utilized data were de-identified in this study.

### Study Design and Population

This study used the Taiwan Cancer Registry (TCR), which is a population-based registry system that includes 90% of all cancer patients in Taiwan (27, 28). We identified patients with advanced lung adenocarcinoma, including those in stages IIIb and IV (M1a and M1b) from the TCR during March 2014 and December 2016. Patients were included if they received gefitinib, erlotinib, or afatinib as first-line treatment within 60 days after diagnosis. Patients were excluded if they received chemotherapy prior to first-line TKI therapy. In Taiwan, gefitinib, erlotinib, and afatinib have been sequentially reimbursed by the Taiwan National Health Insurance (NHI) as first-line therapy for advanced EGFR-mutant lung adenocarcinoma since June 2011, November 2013, and May 2014, respectively (29). Considering that the study period could be an important confounding variable, which could strongly influence the outcome by improving lung cancer treatment, we truncated our dataset to the date of afatinib approval for use (May 2014) in Taiwan.

During the study period, physicians applied for TKI use prior to TKI initiation, and the application was reviewed by the experts of the NHI committee. *EGFR* mutation results, clinical images, pathology, and clinical information were provided along with the application for TKIs. For every 3 months, physicians provided the imaging evidence of partial remission or stable disease to allow further TKI use (https://www.nhi.gov.tw/).

After TKI use, the recruited cohort was then followed, and mortality was confirmed using mortality data from the Department of Statistics, Taiwan. Underlying diseases, TKI use, and duration were ascertained from the Taiwan NHI database (28, 30, 31). Using the linkage between the above-mentioned databases, we longitudinally followed our cohort patients till December 31, 2017.

### Data Collection and Definition

TNM staging data at diagnosis available in the TCR were made according to the International Association for the Study of Lung Cancer 7th edition lung cancer staging system (32). Accordingly, the metastasis (M) category of stage IV lung cancer was subdivided into M1a for cases with intra-thoracic metastases (including pleural seeding, malignant pleural/pericardial effusion, and contralateral pulmonary nodules) and M1b for cases with distant metastases (32). The patients’ performance status was represented as the Eastern Cooperative Oncology Group (ECOG) scores (33). Meanwhile, the Charlson comorbidity index (CCI) was used to assess the patients’ comorbidities using the NHI claims data (34), but malignancy-related score was excluded (cancer-free CCI) as it was previously reported (27). Hospital levels were classified hierarchically into medical centers, regional hospitals, and local hospitals (35). The defining codes for lung adenocarcinoma in the TCR in the NHI are summarized in **Supplementary Table S1**. We also categorized second-line chemotherapy agents into pemetrexed, gemcitabine, paclitaxel, docetaxel, vinorelbine, and others.

### Statistical Analyses

We used proportions or means to describe the demographics and clinical characteristics of the patients. Categorical variables were analyzed using chi-square tests. One-way ANOVA or Student’s t-test was applied for continuous variables. The cohort entry date was that of diagnosis. OS, the primary outcome, was defined as the period from the date of diagnosis to death. Participants were censored if they were still alive at the end of the study period (December 31, 2017). The secondary outcome was time-to-treatment discontinuation (TTD), which was defined as the interval between the date of TKI treatment or chemotherapy initiation and discontinuation. The BMIs were missing for 8% of the patients, but we still considered it important to include BMIs in the final analysis. We imputed the missing values of BMI by age and sex with linear regression methods.

The propensity score (PS) for the probability of TKI administration was derived using a logistic regression model, which included potential confounders such as, age, sex, ECOG, BMI, cancer staging, smoking, alcoholism, CCI, year of TKI use, and hospital level. A 1:1 matched cohort group of afatinib/erlotinib and afatinib/gefitinib was created. Variables that remained significantly different after PS matching were further adjusted in the final model. In this study, only the categorical BMI groups were imbalanced among the different TKI groups, while the absolute values of BMIs were not different between the matched groups.

Subgroup analysis was performed among the different age groups, BMI groups, ECOG groups, sexes, smoking habit, and stages (IIIb, M1a, and M1b). We also compared the OS of five common chemotherapy agents, including pemetrexed, gemcitabine, vinorelbine, paclitaxel, and docetaxel, among the three TKI groups using multivariate Cox regression.

We used SAS (version 9.4; SAS Institute Inc., Cary, NC, USA) for data analyses. A p value of < 0.05 on a two-sided test was considered statistically significant.

## Results

### Demographics and Clinical Variables of the Study

**Figure 1:**
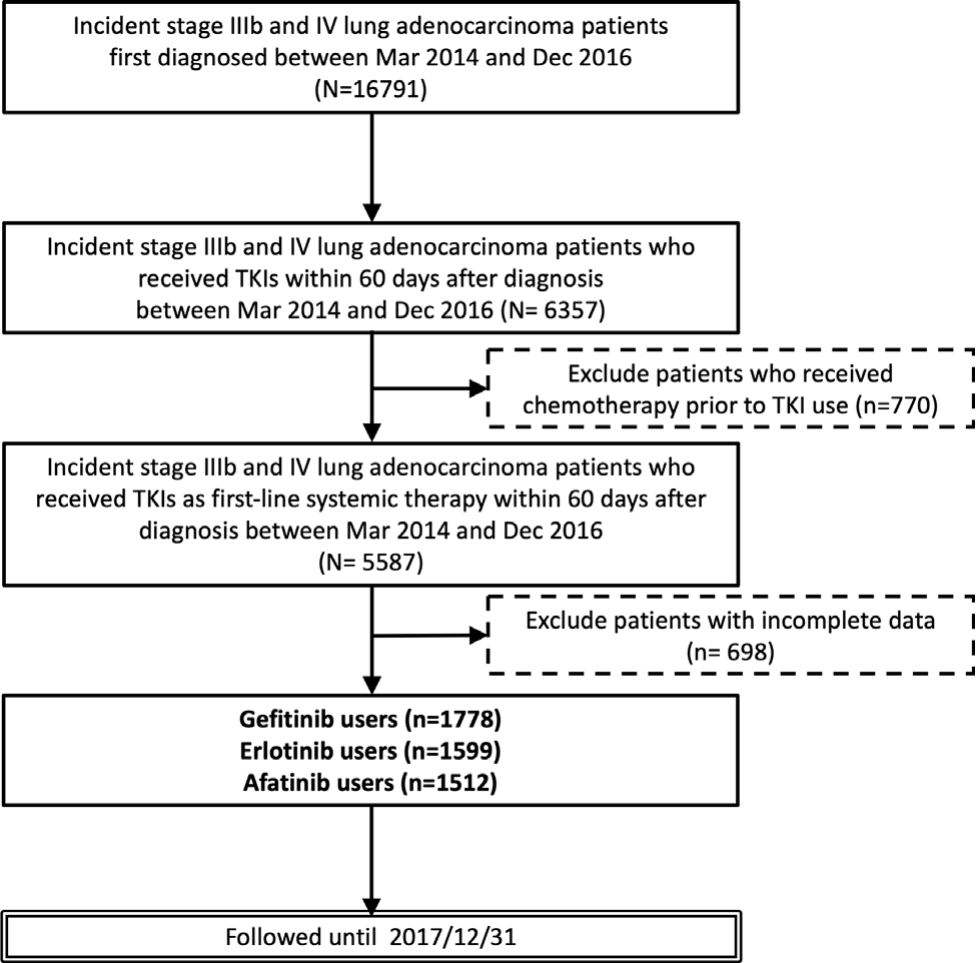
Flow chart of patient recruitment.

Between May 2014 and December 2016, a total of 4,889 advanced lung adenocarcinoma patients with the *EGFR* mutation receiving TKIs (including 1,778 gefitinib, 1,599 erlotinib, and 1,512 afatinib) as first-line therapy were included in our study **(****Figure 1****)**. The baseline characteristics of the enrolled patients are summarized in **Table 1**. The median age of all patients was 67 years, and the majority was female (n = 3,083, 63.1%). Meanwhile, 4,669 (95.5%) patients had stage IV disease. Most eligible patients had relatively good performance status (ECOG ≦ 1: n = 3,780, 77.3%) and had never smoked (n = 3,684, 75.3%).

**Table 1:** Characteristics of advanced lung adenocarcinoma patients receiving epidermal growth factor receptor TKIs as first-line systemic therapy.

	Overall patients(n = 4889)	Gefitinib(n = 1778)	Erlotinib(n = 1599)	Afatinib(n = 1512)	p* value(gefitinib/erlotinib/afatinib)	Erlotinib/Afatinib matched cohort			Gefitinib/Afatinib matched cohort		
						Erlotinib(n = 1228)	Afatinib(n = 1228)	*p* value	Gefitinib(n = 1054)	Afatinib(n = 1054)	*p* value
Age (mean ± SD)	67.3 ± 11.9	70.9 ± 12.0	66.6 ± 11.7	64.4 ± 11.4	<0.0001	65.5 ± 11.6	65.3 ± 11.2	0.6058	66.8 ± 11.9	66.9 ± 10.88	0.8721
<45 years	157 (3.2)	41 (2.3)	50 (3.1)	66 (4.4)	<0.0001	43 (3.5)	35 (2.9)	0.2747	38 (3.6)	22 (2.1)	0.1044
45–65 years	1989 (40.7)	560 (31.5)	677 (42.3)	752 (49.7)		559 (45.5)	595 (48.5)		440 (41.8)	455 (43.2)	
>65 years	2743 (56.1)	1177 (66.2)	872 (54.5)	694 (45.9)		626 (51.0)	598 (48.7)		576 (54.7)	577 (54.7)	
Male, *n* (%)	1806 (36.9)	519 (29.2)	650 (40.7)	637 (42.1)	<0.0001	523 (42.6)	514 (41.8)	0.7131	379 (36.0)	375 (35.6)	0.8558
BMI (mean ± SD)	23.3 ± 3.79	23.0 ± 3.71	23.40 ± 4.10	23.6 ± 3.5	0.0028	23.5 ± 4.3	23.6 ± 3.6	0.6102	23.4 ± 3.5	23.3 ± 3.9	0.6242
<18	262 (5.4)	127 (7.1)	87 (5.4)	48 (3.2)	<0.0001	65 (5.3)	39 (3.2)	0.0265	73 (6.9)	38 (3.6)	0.0023
18–24	3330 (68.1)	1204 (67.7)	1091 (68.2)	1035 (68.5)		824 (67.1)	827 (67.4)		687 (65.2)	726 (68.9)	
>24	1297 (26.5)	447 (25.1)	421 (26.3)	429 (28.4)		339 (27.6)	362 (29.5)		294 (27.9)	290 (27.5)	
Staging, *n* (%)											
IIIb	220 (4.5)	93 (5.2)	58 (3.6)	69 (4.6)	<0.0001	53 (4.3)	52 (4.2)	0.8676	51 (4.8)	49 (4.6)	0.8623
M1a	1415 (28.9)	610 (34.3)	366 (22.9)	439 (29.0)		323 (26.3)	312 (25.4)		342 (32.4)	332 (31.5)	
M1b	3254 (66.6)	1075 (60.5)	1175 (73.5)	1004 (66.4)		852 (69.4)	864 (70.4)		661 (62.7)	673 (63.9)	
ECOG, *n* (%)											
ECOG ≦ 1	3780 (77.3)	1279 (71.9)	1233 (77.1)	1268 (83.9)	<0.0001	1006 (81.9)	996 (81.1)	0.6032	837 (79.4)	844 (80.1)	0.7044
ECOG > 1	1109 (22.7)	499 (28.1)	366 (22.9)	244 (16.1)		222 (18.1)	232 (18.9)		217 (20.6)	210 (19.9)	
Smoking, *n* (%)											
Active smoker	565 (11.6)	171 (9.6)	194 (12.1)	200 (13.2)	0.0031	152 (12.4)	145 (11.81)	0.7434	118 (11.2)	120 (11.4)	0.9587
Ever smoker	640 (13.1)	181 (10.2)	234 (14.6)	225 (14.9)		193 (15.7)	183 (14.9)		125 (11.9)	121 (11.5)	
Never smoker	3684 (75.3)	1426 (80.2)	1171 (73.2)	1087 (71.9)		883 (71.9)	900 (73.3)		811 (76.9)	813 (77.1)	
Alcohol Drinking, *n* (%)											
Active drinker	531 (10.9)	198 (11.1)	165 (10.3)	168 (11.1)	<0.0001	145 (11.8)	142 (11.6)	0.8515	117 (11.1)	108 (10.3)	0.7127
Quitted	203 (4.1)	48 (2.7)	77 (4.8)	78 (5.2)		60 (4.9)	66 (5.9)		38 (3.6)	43 (4.1)	
Never drinker	4155 (85.0)	1532 (86.2)	1357 (84.9)	1266 (83.7)		1023 (83.3)	1020 (83.1)		899 (85.3)	903 (85.7)	
CCI (mean ± SD)	0.79 ± 1.99	0.93 ± 2.11	0.82 ± 2.12	0.60 ± 1.67	<0.0001	0.73 ± 1.83	0.70 ± 1.79	0.9455	0.66 ± 1.79	0.65 ± 1.75	0.6568
Year of use, *n (%)*											
2014	1215 (24.9)	518 (29.1)	450 (28.1)	247 (16.3)	<0.0001	257 (20.9)	228 (18.6)	0.1063	225 (21.4)	215 (20.4)	0.801
2015	1820 (37.2)	708 (39.8)	566 (35.4)	546 (36.1)		428 (34.9)	475 (38.7)		404 (38.3)	417 (39.6)	
2016	1854 (37.9)	552 (31.1)	583 (36.5)	719 (47.6)		543 (44.2)	525 (42.8)		425 (40.3)	422 (40.0)	
Hospital level, *n (%)*											
Medical center	3012 (61.6)	1014 (57.0)	1030 (64.4)	968 (64.0)	<0.0001	795 (64.7)	785 (63.9)	0.9136	643 (61.0)	636 (60.3)	0.9241
Regional hospital	1828 (37.4)	747 (42.0)	552 (34.5)	529 (35.0)		421 (34.3)	431 (35.1)		398 (37.8)	406 (38.5)	
Local hospital	49 (1.0)	17 (1.0)	17 (1.1)	15 (1.0)		12 (1.0)	12 (1.0)		13 (1.2)	12 (1.1)	

BMI, body mass index; CCI, Charlson comorbidity index; ECOG, Eastern Cooperative Oncology Group; SD, standard deviation; TKI, tyrosine kinase inhibitor.

*comparison among three groups (gefitinib, erlotinib, afatinib).

Regarding the comparison among afatinib, gefitinib, and erlotinib users’ characteristics, afatinib users were significantly younger (64.4 ± 11.4 vs. 70.9 ± 12.0 vs. 66.6 ± 11.7 years, p < 0.0001), with higher BMIs (23.6 ± 3.54 vs. 23.0 ± 3.71 vs. 23.4 ± 4.10 kg/m^2^, p = 0.0028) and better ECOG performance status (ECOG ≦ 1, 83.9% vs. 71.9% vs. 77.1%, p < 0.0001), were more active smokers (13.2% vs. 9.6% vs. 12.1%, p = 0.0004), and had lower CCIs (0.60 ± 1.67 vs. 0.93 ± 2.11 vs. 0.82 ± 2.12. p < 0.0001) (**Table 1****)**.

### OS and TTD of Advanced Lung Adenocarcinoma Patients Harboring *EGFR* Mutations and Receiving TKIs as First-Line Therapy

Among all patients, mortality was 48.5% (n = 734) of afatinib, 57.0% (n = 912) of erlotinib, and 62.5% (n = 1112) of gefitinib users. The Kaplan–Meier curve of the three TKIs and OS is illustrated in **Supplementary Figure 1A**. While less than 50% of afatinib users had mortality events, we calculated the OS of users recruited during 2014 and 2015. The OS (mean [median] ± standard deviation, SD) of the gefitinib, erlotinib, and afatinib users recruited during 2014 and 2015 was 20.2 (20) ± 11.7, 20.7 (22) ± 11.7, and 21.8 (24) ± 11.0 months, respectively. The TTD (mean [median] ± SD) of the gefitinib, erlotinib, and afatinib groups was 12.8 (11) ± 9.6, 12.2 (11) ± 9.0, and 13.6 (13) ± 8.9 months, respectively (**Supplementary Figure 1B**).

### Comparing OS and TTD of Matched Afatinib/Erlotinib and Afatinib/Gefitinib Users

In the PS 1:1 matched cohort, two cohorts of 1,228/1,228, afatinib/erlotinib users and 1,054/1,054, afatinib/gefitinib users were assembled. The variables were balanced between the matched groups, while the categorical BMI group remained unbalanced within the groups. The average of BMI, however, remained balanced between the groups (erlotinib vs. afatinib, 23.5 ± 4.3 vs. 23.6 ± 3.6, p = 0.6102; gefitinib vs. afatinib, 23.4 ± 3.5 vs. 23.3 ± 3.9, p = 0.6242) **(****Table 1**).

**Figure 2:**
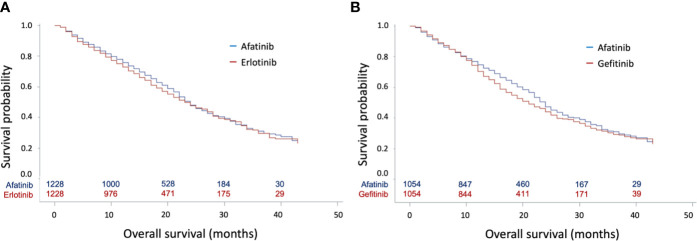
Kaplan–Meier curves for overall survival (OS) according to tyrosine kinase inhibitors (TKIs) in matched cohorts. **(A)** Kaplan–Meier curves for OS between matched afatinib and erlotinib users; **(B)** Kaplan–Meier curves for OS between matched afatinib and gefitinib users.

In the Cox regression analysis, afatinib was not associated with better OS compared with erlotinib (HR: 0.96, 95% CI: 0.86–1.07, p = 0.4673, **Figure 2A**) or gefitinib (HR: 0.91, 95% CI: 0.81–1.02, p = 0.0971, **Figure 2B**). In contrast, patients with afatinib still had longer TTD compared with erlotinib (HR: 0.89, 95% CI: 0.81–0.98, p = 0.0176) (**Supplementary Figure 2A**) and gefitinib (HR: 0.84, 95% CI: 0.76–0.92, p = 0.0004) (**Supplementary Figure 2B**).

### Matched Subgroups Analysis of Afatinib Versus Erlotinib/Gefitinib

**Figure 3:**
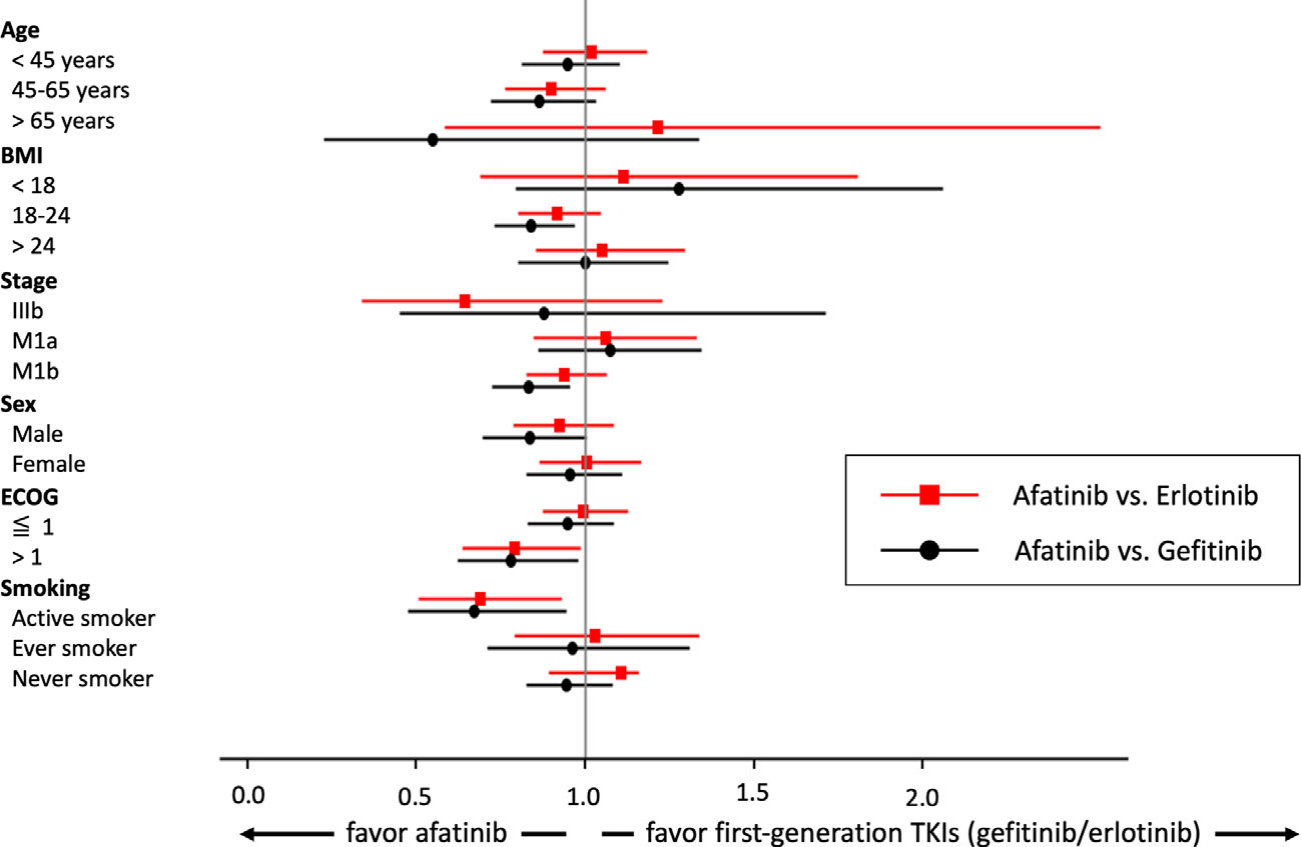
Forest plot for the matched subgroup analysis on overall survival.

A forest plot of the matched subgroups analysis comparing OS between afatinib and first-generation TKIs users is illustrated in **Figure 3**. Interestingly, we found that afatinib use reached statistical significance among the subgroups of active smokers (afatinib vs. erlotinib, HR: 0.69, 95% CI: 0.51–0.93, p = 0.0151; afatinib vs. gefitinib, HR: 0.67, 95% CI: 0.48–0.94, p = 0.022) and ECOG > 1 (afatinib vs. erlotinib, HR: 0.79, 95% CI: 0.63–0.99, p = 0.0375; afatinib vs. gefitinib, HR: 0.78, 95% CI: 0.62–0.98, p = 0.0319). When comparing afatinib and gefitinib, afatinib was also associated with better OS among those with normal BMI (HR: 0.84, 95% CI: 0.73–0.97, p = 0.0149) and M1b staging (HR: 0.83, 95% CI: 0.72–0.95, p = 0.0085).

Meanwhile, TTD between afatinib and first-generation TKI users was also compared in the matched subgroups analysis (**Supplementary Figure 3**). Afatinib use could provide longer TTD among the subgroups of patients aged 45–65 years, with normal BMIs (18–24), M1b staging, female sex, any performance status, and active and non-smokers.

### Subsequent Therapies After First-Line EGFR TKI Treatment

**Figure 4:**
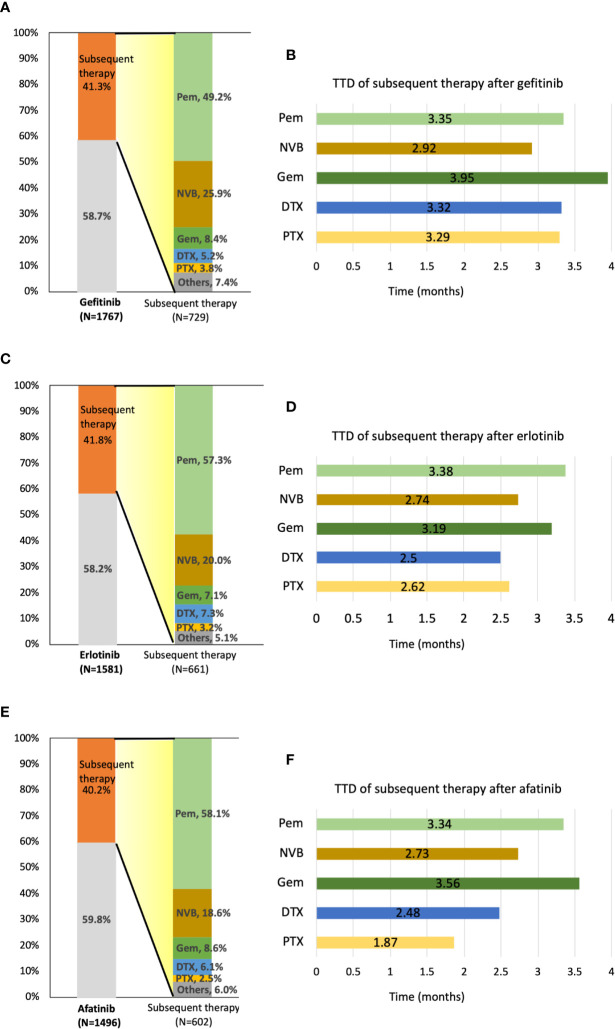
Distribution and time-to-treatment discontinuation (TTD) of second-line chemotherapy agents by different TKIs. **(A)** Percentage of patients who received subsequent therapy and the distribution of second-line treatment agents after gefitinib administration. **(B)** TTD of five second-line treatment agents after gefitinib administration. **(C)** Percentage of patients who received subsequent therapy and the distribution of second-line treatment agents after erlotinib administration. **(D)** TTD of five second-line treatment agents after erlotinib administration. **(E)** Percentage of patients who received subsequent therapy and the distribution of second-line treatment agents after afatinib administration. **(F)** TTD of five second-line treatment agents after afatinib administration. DTX, docetaxel; GEM, gemcitabine; NVB, vinorelbine; Pem, pemetrexed; PTX, paclitaxel.

Forty-five patients, including 16 afatinib, 18 erlotinib, and 11 gefitinib users were still receiving TKI at the end of the follow-up. In total, 1,992 of 4,844 patients (41.1%) received subsequent treatment as second-line therapy. A total of 729 patients (41.3%) in the gefitinib group, 661 patients (41.8%) in the erlotinib group, and 602 (40.2%) in the afatinib group received subsequent chemotherapy after receiving EGFR TKIs **(****Figure 4****)**. Of the 1,992 patients receiving subsequent chemotherapy, 1,120 patients (56.2%) received platinum-based doublets as treatment, i.e., 359 patients (49.2% of 729) in the gefitinib group, 372 patients (56.3% of 661) in the erlotinib group, and 389 (64.6% of 602) in the afatinib group (**Supplementary Table S2**). Pemetrexed (1,088 of 1,992 patients, 54.6%) constituted the majority of second-line regimens, followed by vinorelbine (n = 433, 21.7%), gemcitabine (n = 160, 8.0%), docetaxel (n = 123, 6.2%), and paclitaxel (n = 64, 3.2%). Pemetrexed (76.9%) and gemcitabine (50.6%) were the most common partners for platinum drugs, and only 18.5% of patients with vinorelbine simultaneously received platinum drugs (**Supplementary Table S2****)**. For subsequent therapy in subgroup analyses, patients who received erlotinib and afatinib as first-line treatment had a significantly higher proportion of pemetrexed usage (n = 379, 57.3%, p = 0.0025 and n = 350, 58.1%, p = 0.0012, respectively) as second-line therapy compared to the gefitinib group (n = 359, 49.2%). Among gefitinib users, a higher proportion of patients received vinorelbine (n = 189, 25.9%) than that in the erlotinib and afatinib groups (n = 132, 20.0%, p = 0.0085 and n = 112, 18.6%, p = 0.0015, respectively) **(****Figure 4**).

### OS Among Different Second-Line Systemic Chemotherapy After First-Line EGFR TKI Treatment

After first-line TKI therapy, the TTD of subsequent systemic chemotherapy was around 2.7–3.6 months (pemetrexed: 3.36 ± 3.53 months; vinorelbine: 2.82 ± 4.23 months; gemcitabine: 3.60 ± 5.56 months; docetaxel: 2.74 ± 3.08 months; paclitaxel 2.73 ± 2.96 months) (**Supplementary Table S3****)**. As second-line therapy, pemetrexed and gemcitabine both have longer TTD compared with other chemotherapy agents, regardless of first-line TKI agents (**Figure 4**).

Comparing the OS of different second-line chemotherapy regimens, pemetrexed was associated with better OS than was vinorelbine in gefitinib users with advanced *EGFR* mutant lung adenocarcinoma (Ref: pemetrexed, HR: 1.65, 95% CI: 1.28–2.13, p = 0.0001) (**Table 2**). In erlotinib users, pemetrexed showed superiority in the longest TTD of all regimens as second-line treatment. Pemetrexed and gemcitabine had similar OS, which was longer than that of vinorelbine, docetaxel, or paclitaxel among afatinib users.

**Table 2:** Comparison of the overall survival of five common chemotherapy regimens as a subsequent therapy of the three EGFR TKI groups.

	Gefitinib			Erlotinib			Afatinib		
	Hazard ratio	95% CI	*p* value	Hazard ratio	95% CI	*p* value	Hazard ratio	95% CI	*p* value
Pemetrexed	Ref			Ref			Ref		
Gemcitabine	1.26	0.86–1.85	0.239	1.87	1.20–2.90	0.0053	1.37	0.90–2.09	0.146
Vinorelbine	1.65	1.28–2.13	0.0001	1.59	1.19–2.12	0.0019	1.67	1.21–2.32	0.0021
Docetaxel	1.50	0.96–2.36	0.0767	1.61	1.08–2.41	0.0191	2.08	1.37–3.16	0.0006
Paclitaxel	1.44	0.86–2.41	0.1627	1.81	1.01–3.25	0.0463	2.24	1.15–4.33	0.0173

CI, confidence interval.

## Discussion

Our study showed that afatinib did not provide the evidence of a survival advantage over gefitinib and erlotinib. In the subgroup analysis, afatinib was associated with better OS among patients with active smoking and poor performance status. While 40% of patients were able to receive second-line chemotherapy agents, pemetrexed was associated with better OS across the three TKI groups. An alternative choice may be gemcitabine.

To the best of our knowledge, this study is the single largest cohort study to investigate the effectiveness of three EGFR TKIs (16, 18). We found that compared with first-generation TKIs, patients receiving afatinib were younger, were more likely to be male, had higher BMIs, and had better performance status. Previous studies have shown that afatinib provided longer PFS than first-generation TKIs (15, 18, 20), but adverse effects in patients receiving afatinib were also more frequently observed (15). In real-world practice, our research showed that the baseline characteristics could significantly influence the clinicians’ judgments and preferences while choosing one of the TKIs (gefitinib, erlotinib, or afatinib) as first-line treatment. Afatinib may be preferred among those who are younger, are male, have higher BMIs, are active smokers, and have better performance status. Interestingly, we found that the proportion of afatinib users among all TKI users had increased in the recent years. In 2016, the number of afatinib users surpassed either erlotinib or gefitinib users. As physicians became experienced in managing patients with afatinib (especially the toxicity profiles), they selected afatinib over erlotinib or gefitinib.

In PS matching analysis, afatinib use was not associated with better OS compared with erlotinib or gefitinib use. In the subgroup analysis, afatinib use was associated with survival benefit among patients with ECOG > 1 and active smoking. While one may argue that failure to demonstrate the clinical evidence of survival benefit may be due to the relatively small sample size in previous randomized control trials and other observational studies, our study may have the current largest cohort, including more than 1,000 participants in each TKI group (14, 16, 17, 36, 37). More importantly, we used PS matching, which is a more robust way of controlling confounders in observational studies, and this analysis strategy was not performed in previous observational studies (38).

Clinically, afatinib could be an effective treatment for lung adenocarcinoma patients with the *EGFR* mutation and brain metastasis (39). Subgroup analysis showed that afatinib provided better OS in patients with distant metastases (stage M1b) compared with gefitinib, but this benefit was not observed when compared with the erlotinib group. Active/current smokers usually have lower *EGFR* mutation rates (especially exon 19 deletion and p.L858R) than never-smoking female patients (8, 40). Notably, our study (through subgroup analysis) showed that afatinib could provide significantly longer OS in active smokers than could gefitinib/erlotinib. The above-mentioned benefits may be because afatinib is a member of the pan-ErbB family of inhibitors. It could covalently and irreversibly bind to the intracellular tyrosine kinase domain of the EGFR and effectively treat common (exon 19 deletion and p.L858R) and uncommon *EGFR* mutations (41–43). Furthermore, this real-world study provided additional information on the minor population with a poor performance status (ECOG >1), which is often excluded by randomized controlled trials to evaluate the efficacy and safety of the study drugs. None of the patients (among the 310 patients) with ECOG > 1 in the phase IIb LUX-Lung 7 trial (afatinib vs. gefitinib) were enrolled (19), and only 6 (2.3%) patients with ECOG = 2 (among 256 patients) in the phase III CTONG 0901 trial (gefitinib vs. erlotinib) were included (13). In contrast, there were 1,109 patients (up to 22.7% of total 4,889 patients) with ECOG > 1 in real-world practice, and afatinib surprisingly demonstrated superior TTD and OS benefits compared with first-generation TKIs in patients with worse performance status.

Approximately 40%–60% of acquired *EGFR* p.T790M develops after the patients receive first-line TKIs (44, 45), and osimertinib was approved as second-line treatment by the US Food and Drug Administration (FDA) and Taiwan Food and Drug Administration in November 2015 and November 2016, respectively. In the FLAURA trial, 14.1% (39 of 277) of patients received chemotherapy and 30.7% (85 of 277) of patients in the gefitinib/erlotinib group received osimertinib as second-line treatment (46). Osimertinib, however, was not reimbursed by the NHI during the study period. Shifting to osimertinib treatment after first-line TKI failure, therefore, was not widely used during our study period in Taiwan, and platinum-based doublets remained the standard for second-line treatment. Our real-world study indicated that 41.1% of all TKI patients in Taiwan could have subsequent cytotoxic chemotherapy as an effective treatment. While chemotherapy may be the most important and preferred systemic therapy after the failure of first-line osimertinib treatment, only 32.3% (90 of 279) of patients in the osimertinib group of the FLAURA trial received chemotherapy as a subsequent systemic therapy (46). In our study, pemetrexed (54.6%) and vinorelbine (21.7%) were the most common subsequent chemotherapy agents. Pemetrexed was the most preferred subsequent therapy in clinical practice owing to its efficacy, tolerability, and convenience in administration (47), and vinorelbine was also frequently prescribed because of the oral route of administration and less toxicity in elderly patients (48). In this real-world study, only 56.2% of patients used platinum-based doublets as subsequent chemotherapy agents after TKI failure. Interestingly, pemetrexed and gemcitabine were found to be the most common partners for platinum drugs. Meanwhile, pemetrexed as a subsequent therapy could provide the best TTD benefit among all agents in erlotinib users. Furthermore, pemetrexed and gemcitabine demonstrated similar effectiveness in TTD among gefitinib and afatinib users. These findings from our claims database epidemiological studies could provide personalized guidance in clinical practice, complementary to biomarker and genetic risk factor studies for oncological patients.

There were some limitations in our study. First, detailed information on *EGFR* mutation sites was not available in the TCR database. Therefore, the effectiveness of different generation TKIs could not be compared with common or uncommon mutations. Meanwhile, the causes of TTD and TKI-related toxicity profiles could not be readily clarified. In the subsequent treatment analysis, osimertinib was not reimbursed by the Taiwan NHI. Therefore, self-financed or clinical trial osimertinib users could not be identified in this study. Finally, the FLAURA trial demonstrated the superior efficacy and safety of osimertinib compared with gefitinib and erlotinib as first-line TKIs in *EGFR* mutant NSCLC patients, and osimertinib is therefore currently considered the standard for first-line therapy (46). However, data regarding the activity of osimertinib in patients harboring rare *EGFR* mutations are limited. Economic issues, such as high cost and the lack of insurance reimbursement may preclude osimertinib use in real-world. Meanwhile, the optimal therapeutic strategy for disease progression after osimertinib administration may still be ambiguous for physicians because of the lack of large-scale real-world data. Gefitinib, erlotinib, and afatinib are, therefore, still used as first-line treatment in many *EGFR* mutant NSCLC patients.

Our study indicates that despite the increasing use of afatinib as first-line TKI for *EGFR* mutant, late-stage adenocarcinoma patients, afatinib use was not associated with longer OS than were first-generation TKIs. Afatinib administration, however, may be considered among active smokers. Additionally, for patients with poor performance status, afatinib administration may also lead to survival benefits and should not be withheld due to the fear of toxicity. For second-line chemotherapy, pemetrexed may be the preferred agent, and gemcitabine can also be considered as a reasonable alternative.

## Data Availability Statement

All datasets presented in this study are included in the article/**Supplementary Material**.

## Ethics Statement

The studies involving human participants were reviewed and approved by Institutional Review Board (IRB) committee of National Taiwan University Hospital Hsinchu Branch. Written informed consent for participation was not required for this study in accordance with the national legislation and the institutional requirements.

## Author Contributions

M-RL and S-KL designed all experiments. M-RL, S-KL, C-HC, Y-FW, and L-TK conducted the experiments and analyzed and interpreted the results. C-YY, J-YS, J-YW, J-CK, C-YY, and C-JY supervised the project. M-RL, C-HC, and S-KL wrote the draft manuscript. C-YY, J-YS, J-YW, J-CK, C-YY, and C-JY reviewed and edited the manuscript. All authors contributed to the article and approved the submitted version.

## Funding

This work was supported by the National Taiwan University Hospital Hsinchu Branch Research Grant NTUH-HC 106-HCH022 and NTUH-HC 108-s267.

## Conflict of Interest

S-KL has received honoraria for speeches from Roche, AstraZeneca, Pfizer, Merck Sharp & Dohme, Novartis, and Boehringer Ingelheim. M-RL has received honoraria for speeches from Roche, Pfizer, and Daiichi Sankyo. J-YS has received speaking honoraria from AstraZeneca, Roche, Boehringer Ingelheim, Pfizer, Novartis, Bristol-Myers Squibb, Merck Sharp & Dohme, and Eli Lilly, and has been paid for fulfilling a consulting or advisory role by AstraZeneca, Roche, Boehringer Ingelheim, Novartis, Merck Sharp & Dohme, AbbVie, and Chugai Pharmaceutical.

The remaining authors declare that the research was conducted in the absence of any commercial or financial relationships that could be construed as a potential conflict of interest.

## Supplementary Material

Supplementary Figure 1 Kaplan–Meier curves for overall survival (OS) and time-to-treatment discontinuation (TTD) for 3 tyrosine kinase inhibitors (TKIs). **(A)** Kaplan–Meier curves of OS for afatinib, erlotinib, and gefitinib administration; **(B)** Kaplan–Meier curves of TTD for afatinib, erlotinib, and gefitinib administration.

Supplementary Figure 2 **(A)** Kaplan–Meier curves of time-to-treatment discontinuation (TTD) between matched afatinib and erlotinib groups; **(B)** Kaplan–Meier curves for TTD between matched afatinib and gefitinib groups

Supplementary Figure 3 Forest plot for the matched subgroup analysis on time-to-treatment discontinuation.

## Abbreviations

BMI, body mass index; CI, confidence interval; ECOG, Eastern Cooperative Oncology Group; EGFR, epidermal growth factor receptor; HR, hazard ratio; NHI, National Health Insurance; OS, overall survival; PFS, progression-free survival; SD, standard deviation; TKI, tyrosine kinase inhibitor; TTD, time-to-treatment discontinuation.
