# Supplementary material for: Treatment Options of First-Line Tyrosine Kinase Inhibitors and Subsequent Systemic Chemotherapy Agents for Advanced EGFR Mutant Lung Adenocarcinoma Patients: Implications From Taiwan Cancer Registry Cohort
Source: Front Oncol. 2021 Jan 8;10:590356. doi: 10.3389/fonc.2020.590356 (PMC7821751; doi:10.3389/fonc.2020.590356)
Supplement: Supplementary file 1 [file DataSheet_1.docx]

**Supplementary Table S1:** Defining codes in this study for Taiwan Cancer Registry and National Health Insurance database

| **Taiwan Cancer Registry** | **Definition** |
| --- | --- |
| Lung adenocarcinoma | ICD-O-3 code International Classification of Diseases for Oncology, 3rd edition (ICD-O-3) code for lung site (C340-C343,C348-C349) plus adenocarcinoma (8140, 8141, 8143, 8147,8200, 8201,8250~8255, 8260, 8310, 8320, 8323, 8480, 8481, 8490) |

**Supplementary Table S2:** Detailed information of patient distribution in different subsequent regimens

|  | | **Total patients** | | **Gefitinib users** | | **Erlotinib users** | | **Afatinib users** | |
| --- | --- | --- | --- | --- | --- | --- | --- | --- | --- |
| Subsequent treatment (with platinum, %) | | 1992 (1120, 56.2%) | | 729 (359, 49.2%) | | 661 (372, 56.3%) | | 602 (389, 64.6%) | |
| Pemetrexed (with platinum, %) | | 1088 (837, 76.9%) | | 359 (273, 76.0%) | | 379 (276, 72.8%) | | 350 (288, 82.3%) | |
| Vinorelbine (with platinum, %) | | 433 (80, 18.5%) | | 189 (25, 13.2%) | | 132 (33, 25.0%) | | 112 (22, 19.6%) | |
| Gemcitabine (with platinum, %) | | 160 (81, 50.6%) | | 61 (24, 39.3%) | | 47 (23, 48.9%) | | 52 (34, 65.4%) | |
| Docetaxel (with platinum, %) | | 123 (52, 42.3%) | | 38 (13, 34.2%) | | 48 (19, 39.6%) | | 37 (20. 54.1%) | |
| Paclitaxel (with platinum, %) | | 64 (25, 39.1%) | | 28 (7, 25.0%) | | 21 (11, 52.4%) | | 15 (7, 46.7%) | |
| Others (with platinum, %) | | 124 (45, 36.3%) | | 54 (17.31.5%) | | 34 (10, 29.4%) | | 36 (18, 50.0%) | |

Abbreviation: TKI, tyrosine kinase inhibitor.

**Supplementary Table S3:** Time-to-treatment discontinuation of individual chemotherapy regimen as subsequent therapy

| Chemotherapy regimen | Total patients | Gefitinib users | Erlotinib users | Afatinib users |
| --- | --- | --- | --- | --- |
| Pemetrexed, TTD (mean ± SD, months) | 3.36 ± 3.53 | 3.35 ± 3.76 | 3.38 ± 3.75 | 3.34 ± 3.02 |
| Vinorelbine, TTD (mean ± SD, months) | 2.82 ± 4.23 | 2.92 ± 4.32 | 2.74 ± 4.28 | 2.73 ± 4.04 |
| Gemcitabine, TTD (mean ± SD, months) | 3.60 ± 5.56 | 3.95 ± 4.88 | 3.19 ± 5.90 | 3.56 ± 6.04 |
| Docetaxel, TTD (mean ± SD, months) | 2.74 ± 3.08 | 3.32 ± 3.80 | 2.50 ± 3.11 | 2.48 ± 2.06 |
| Paclitaxel, TTD (mean ± SD, months) | 2.73 ± 2.96 | 3.29 ± 3.48 | 2.62 ± 2.33 | 1.87 ± 2.58 |

Abbreviation: SD, standard deviation; TTD, time-to-treatment discontinuation.
