# Supplementary figures and images for: Treatment Options of First-Line Tyrosine Kinase Inhibitors and Subsequent Systemic Chemotherapy Agents for Advanced EGFR Mutant Lung Adenocarcinoma Patients: Implications From Taiwan Cancer Registry Cohort
Source: Front Oncol. 2021 Jan 8;10:590356. doi: 10.3389/fonc.2020.590356 (PMC7821751; doi:10.3389/fonc.2020.590356)

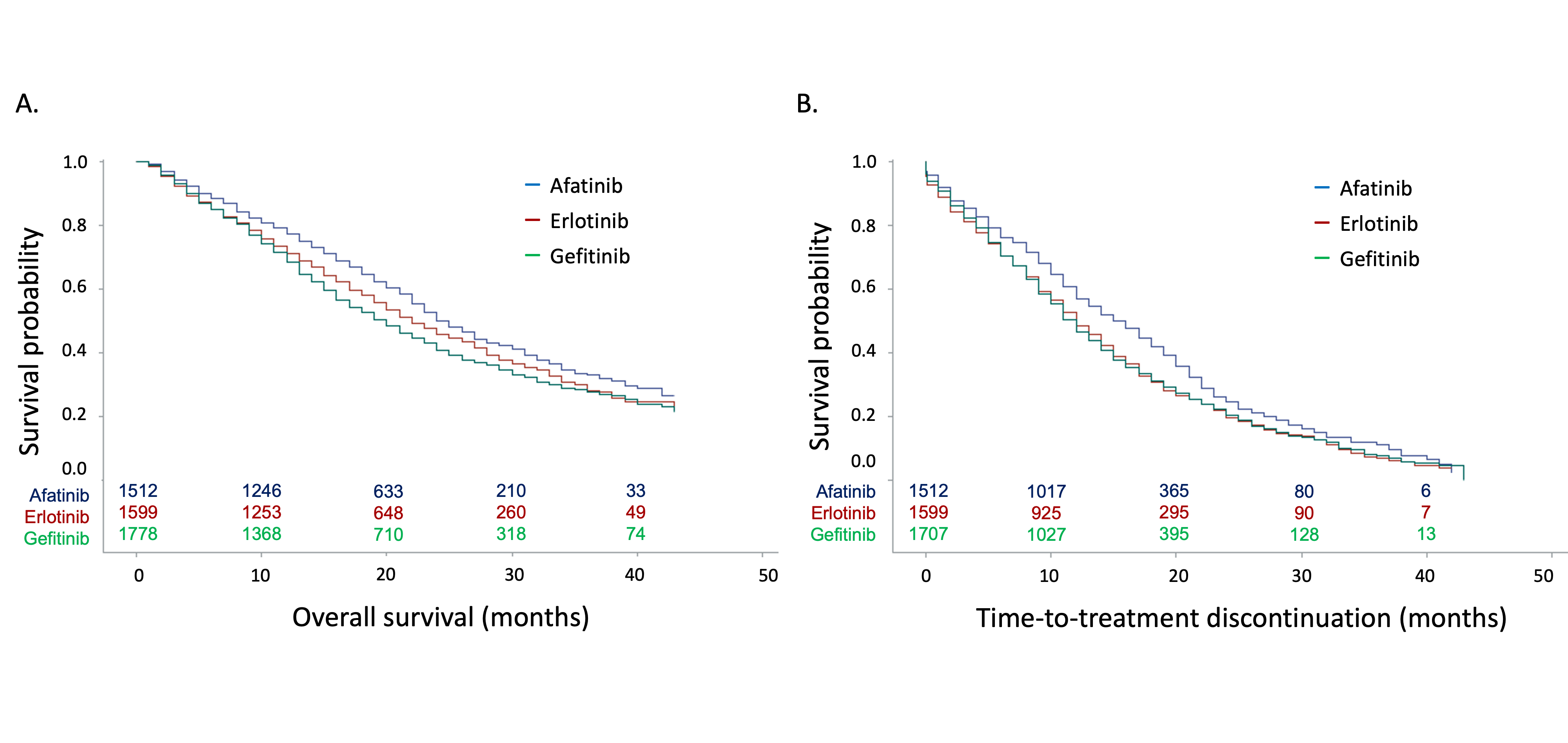

Supplement: Supplementary Figure 1 — Kaplan–Meier curves for overall survival (OS) and time-to-treatment discontinuation (TTD) for 3 tyrosine kinase inhibitors (TKIs). (A) Kaplan–Meier curves of OS for afatinib, erlotinib, and gefitinib administration; (B) Kaplan–Meier curves of TTD for afatinib, erlotinib, and gefitinib administration. [file Image_1.tiff]

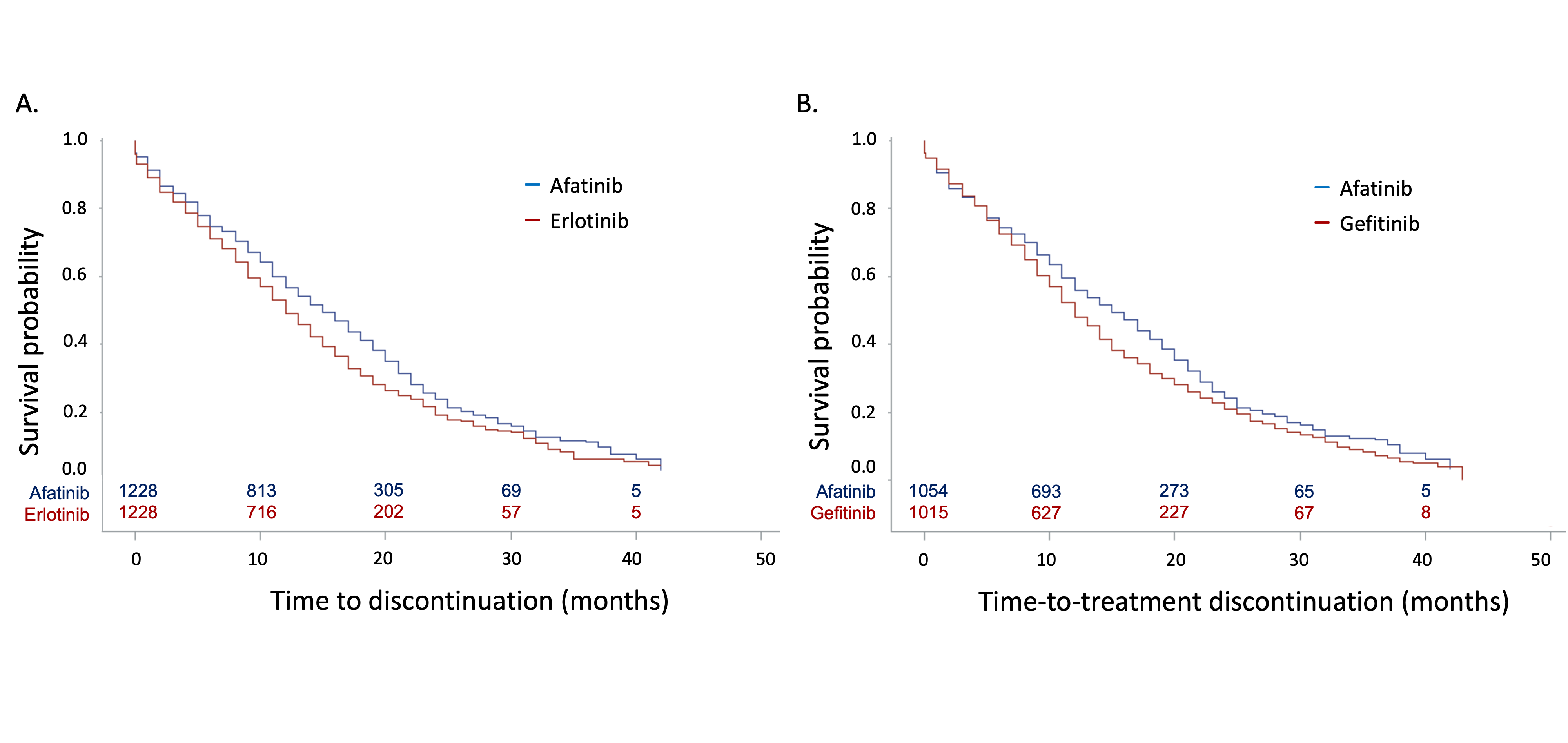

Supplement: Supplementary Figure 2 — (A) Kaplan–Meier curves of time-to-treatment discontinuation (TTD) between matched afatinib and erlotinib groups; (B) Kaplan–Meier curves for TTD between matched afatinib and gefitinib groups [file Image_2.tiff]

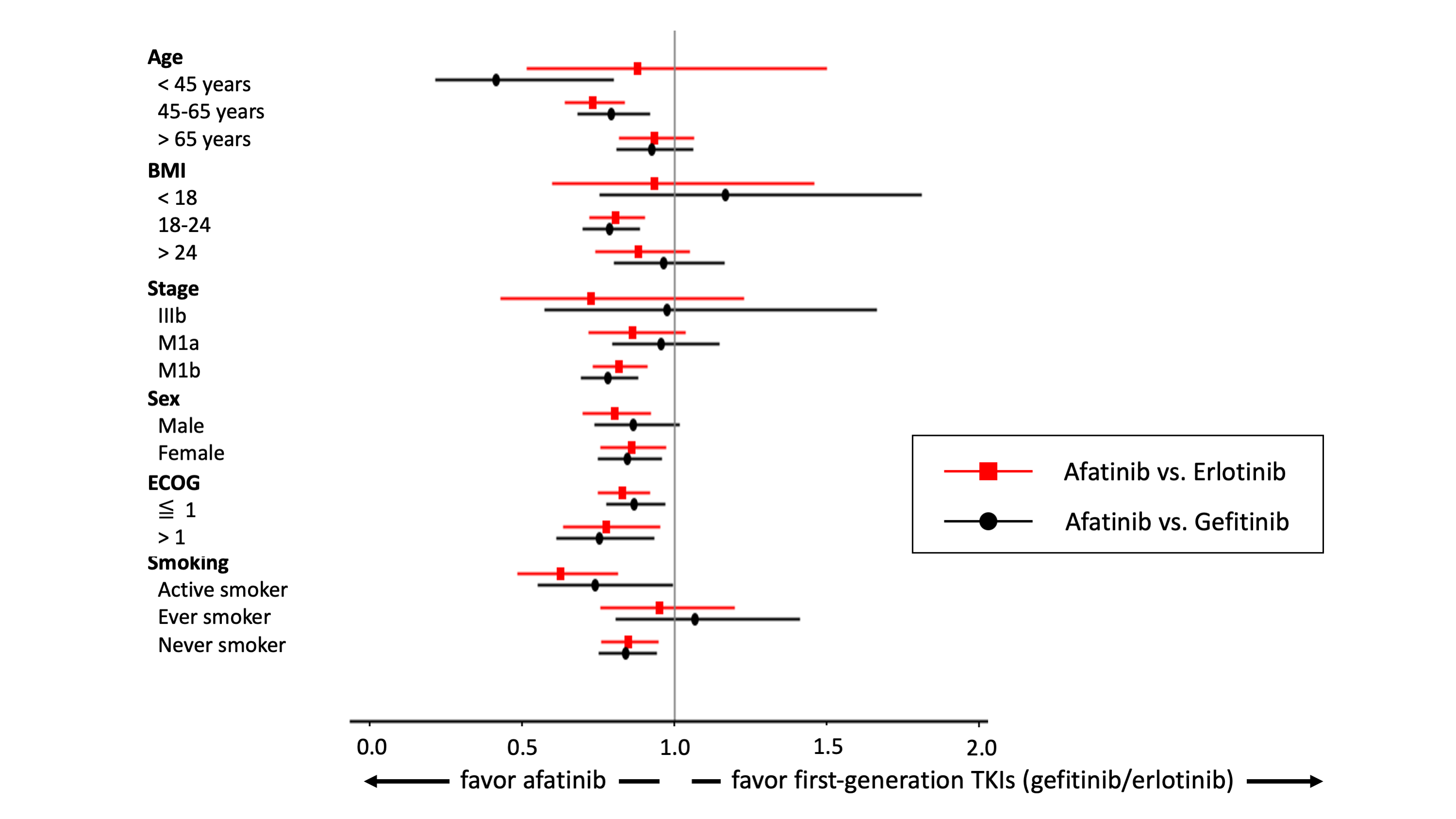

Supplement: Supplementary Figure 3 — Forest plot for the matched subgroup analysis on time-to-treatment discontinuation. [file Image_3.tiff]
